# Impact of manual correction over automated segmentation of spectral domain optical coherence tomography

**DOI:** 10.1186/s40942-020-0207-6

**Published:** 2020-02-14

**Authors:** Alexandre Gomes Bortoloti de Azevedo, Guilherme Eiichi da Silva Takitani, Bruno Rebello Godoy, Bruna Ferraço Marianelli, Vinicius Saraiva, Ivan Maynart Tavares, Luiz Roisman

**Affiliations:** Departament of Ophthalmology and Visual Sciences, Federal University of São Paulo-UNIFESP/EPM, Rua Pedro de Toledo, 781, São Paulo, SP 04039-032 Brazil

**Keywords:** OCT, Segmentation, Manual correction

## Abstract

**Objective:**

To study the automated segmentation of retinal layers using spectral domain optical coherence tomography (OCT) and the impact of manual correction over segmentation mistakes.

**Methods:**

This was a retrospective, cross-sectional, comparative study that compared the automated segmentation of macular thickness using Spectralis™ OCT technology (Heidelberg Engineering, Heidelberg, Germany) versus manual segmentation in eyes with no macular changes, macular cystoid edema (CME), and choroidal neovascularization (CNV). Automated segmentation of macular thickness was manually corrected by two independent examiners and reanalyzed by them together in case of disagreement.

**Results:**

In total, 306 eyes of 254 consecutive patients were evaluated. No statistically significant differences were noted between automated and manual macular thickness measurements in patients with normal maculas, while a statistically significant difference was found in central thickness in patients with CNV and with CME. Segmentation mistakes in macular OCTs were present in 5.3% (5 of 95) in the normal macula group, 16.4% (23 of 140) in the CME group, and 66.2% (47 of 71) in CNV group. The difference between automated and manual macular thickness was higher than 10% in 1.4% (2 of 140) in the CME group and in 28.17% (20 of 71) in the CNV group. Only one case in the normal group had a higher than 10% segmentation error (1 of 95).

**Conclusion:**

The evaluation of automated segmented OCT images revealed appropriate delimitation of macular thickness in patients with no macular changes or with CME, since the frequency and magnitude of the segmentation mistakes had low impact over clinical evaluation of the images. Conversely, automated macular thickness segmentation in patients with CNV showed a high frequency and magnitude of mistakes, with potential impact on clinical analysis.

## Introduction

Optical coherence tomography (OCT) is a widely used noninvasive diagnostic device that provides objective evaluation and measurements of retinal structures, such as macular thickness, through automated segmentation of macular layers [1]. It is largely used to define the criteria of progression, stabilization, or regression of diseases. OCT results have been used in various studies and clinical trials as the inclusion and therapeutic response criteria of many retinal treatments. Consequently, OCT has a strong influence on the decisions for maintaining, interrupting, or changing therapy, especially in common conditions, such as age related macular degeneration (AMD) and diabetic macular edema [2–5].

The Spectralis™ OCT has tools for manual correction of automated segmentation, and previous studies have shown significant differences between the automated and the manual measurements in macular pathologies [6–8]. These differences could possibly influence decision-making, so recognizing the presence of automated segmentation errors, and knowing in which pathologies these errors are more common is important. In this study, we compared the frequency and the magnitude of the errors of Spectralis™ OCT automated segmentation to manual segmentation for normal eyes, eyes with cystoid macular edema (CME), and eyes with choroidal neovascularization (CNV).

## Materials and methods

This retrospective, cross-sectional, comparative study was conducted at the Retina Service of the Federal University of São Paulo, after approval from the university’s investigational review board.

### Participants

Patients with normal macular anatomy, CME, and CNV were selected and retrospectively analyzed. Images were excluded if they did not include the whole retinal thickness or present other coexisting ocular conditions, such as retinoschisis, posterior pole staphyloma, chorioretinal scars, macular hole, epiretinal membrane, and other posterior pole lesions.

### OCT

Heidelberg Spectralis OCT (Heidelberg Eye Explorer version 1.9.10.0, HRA2 Family acquisition module 6.3.2.0, Viewing module 6.3.4.0) images included either 25 or 39 horizontal raster lines (Fast mode). Retinal thickness measurements of the center of the fovea [central subfield of nine early treatment diabetic retinopathy study (ETDRS) subfields] were acquired and analyzed before and after manual correction.

### Image quality

Each OCT examination was assessed using the mean quality index (mean QI) of all its images and by the minimal quality index (lowest quality index image = Min QI). No exams were excluded due to low QI. Segmentation error frequencies and QI index correlations were assessed by statistical analysis.

### Measurements errors determination and manual segmentation correction

Automatized segmentation was compared to manual correction accomplished by two independent examiners (A.A., B.M.). In cases of disagreement, both readers reanalyzed the images and a consensus was obtained.

**Fig. 1:**
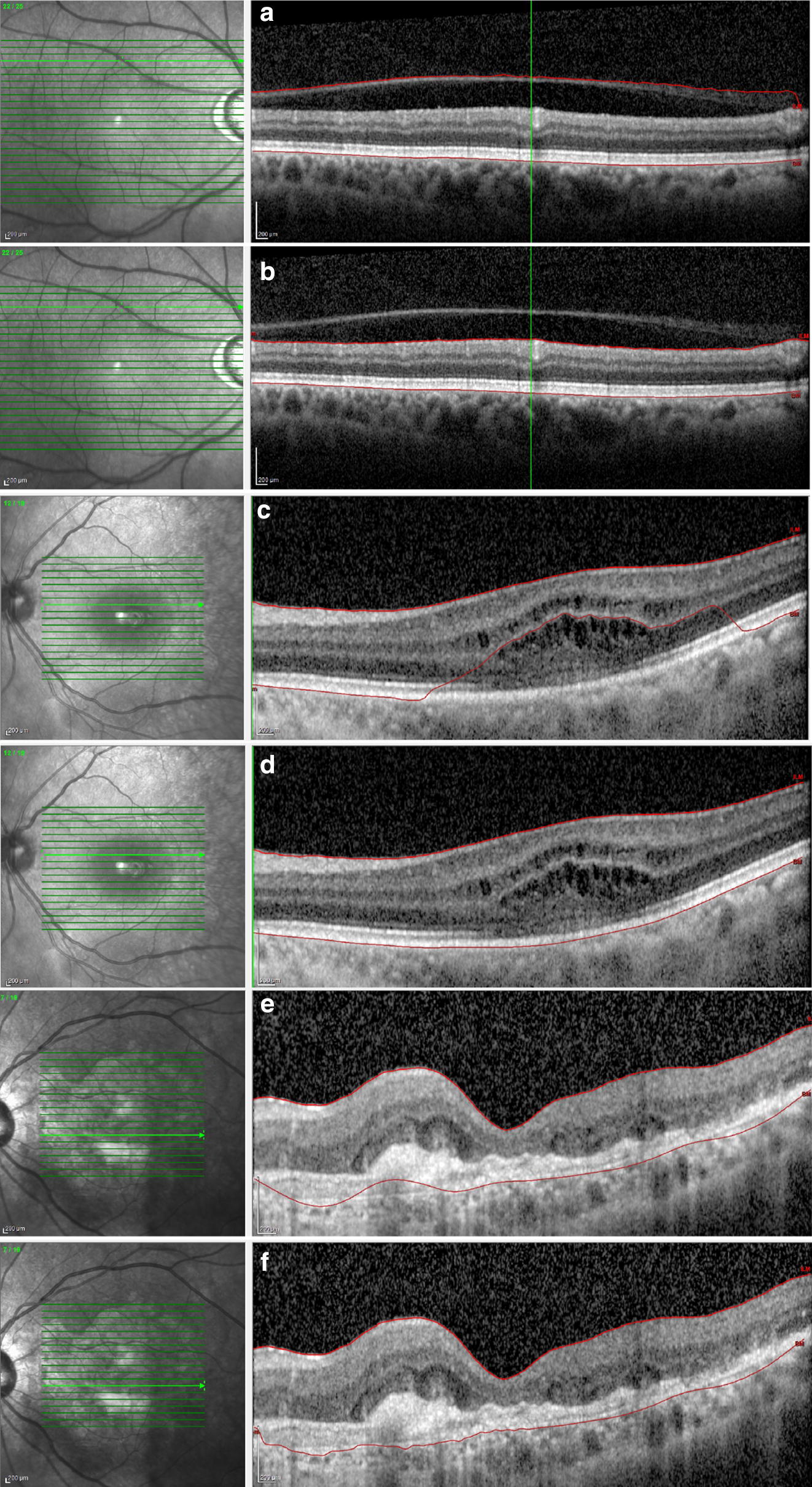
Examples of segmentation error and the manual correction of (**a**, **b**) the internal limiting membrane in a normal macula and of the Bruch’s membrane in (**c**, **d**) an eye with cystoid macular edema and (**e**, **f**) choroidal neovascularization

The images provided by the software for the three groups were analyzed, and the pattern of the mistakes was identified: internal limiting membrane (ILM) delineation error, Bruch’s membrane (BM) delineation error, or both. The segmentation of each b-scan of the raster scan was then manually corrected to the proper position (Fig. 1). A variation higher than 10% in the ETDRS thickness map was considered a clinically relevant error. This criterion was based on protocols that established a variance of 10% in the central subfield thickness as a therapeutic response when treating macular edema with anti-VEGF drugs [3–5].

### Statistical analysis

The difference in the frequency of significant mistakes (greater than 10%) in the groups was analyzed by Student’s t test.

Statistical analysis was conducted using specific statistical software (SAS University Edition, 2016, Cary, NC, USA). In each group, the subfield thicknesses were measured before and after manual correction of delimitation.

The Shapiro–Wilk test was used to determine if the values had a normal distribution. The measurement differences were analyzed by a paired t test for normal distributions and by the Wilcoxon Signed Rank for skewed distributions. A p value < 0.05 was considered statistically significant.

## Results

In total, 372 OCT scans of 254 patients imaged between August 2015 and February 2016 were reviewed. Overall, 66 images were excluded, 52 exams due to incomplete retinal scanning and 14 exams due to concomitant macular diseases. From those, a total of 306 images of 241 patients were included and divided into three groups: 95 in the normal macula group, 140 in the CME group, and 71 in the CNV group. The mean age was 53.75 years in the normal macula group, 60.43 years in the CME group, and 71.15 years in the CNV group.

In the normal macula group, the mean difference of central thickness between automatic and manual measurements was 9.80 ± 15.01 μm, with no statistically significant difference. This difference was 14.83 ± 30.87 μm in the CME group (p = 0.002) and 52.55 ± 63.467 μm in the CNV group (p < 0.0001).

The analysis between mean central error of the different groups revealed a higher magnitude of error in the CNV group (p < 0.001). No significant difference was noted between the magnitudes of the mean central error when comparing the CME and normal macula groups.

**Fig. 2:**
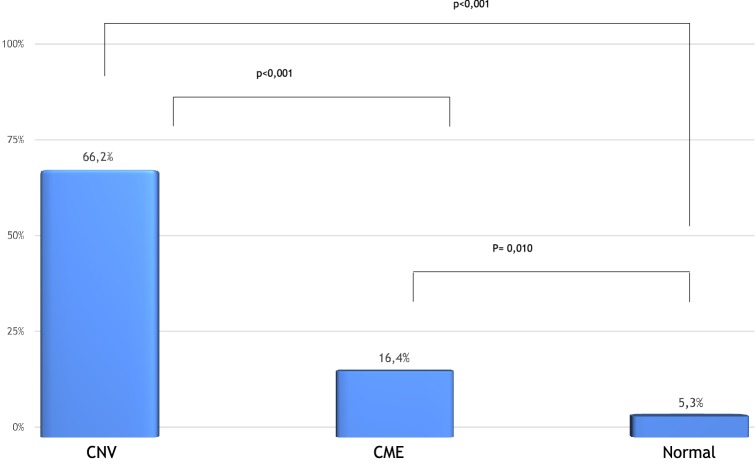
Comparison of the frequency of segmentation errors between the normal macula, the cystoid macular edema (CME), and the choroidal neovascularization (CNV) groups

The frequency of segmentation errors was 5.3% (5 of 95) in the normal macula group, 16.4% (23 of 140) in the CME group, and 66.2% (47 of 71) in the CNV group. The frequency of errors between the three groups was statistically different (p < 0.001). The error frequency was significantly higher in CNV group than in the CME group, whereas the errors were statistically higher in the CME group than in the normal macula group (Fig. 2).

**Fig. 3:**
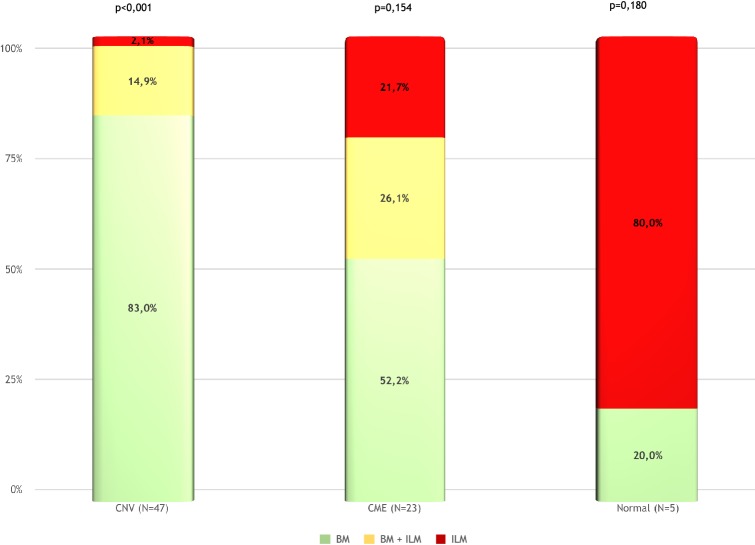
Comparison of the location of the segmentation errors among the normal macula, the cystoid macular edema (CME), and the choroidal neovascularization (CNV) groups. *BM* Bruch’s membrane, *ILM * internal limiting membrane

In the normal macula group, the most common error affected the ILM delimitation, which occurred in four of five cases. In three of those four cases, this happened due to the delimitation of the posterior hyaloid, as it was the ILM. In one case, there was an isolated error of BM delineation. For the CME group, the concomitant error of BM and ILM occurred in 7 of 23 cases (30.43%), an isolated BM error in 11 of 23 cases (47.82%), and an isolated ILM inaccuracy in 5 cases (21.73%). For the CNV group, the concomitant error of BM and ILM occurred in 7 of 47 cases (14.89%) and an isolated BM error in 39 (82.97%) of the eyes. Thus, 97.87% of the errors evolved BM. Only one case showed an isolated ILM error in the CNV group. The distribution of error location was not homogenous for the CNV group (p < 0.001) (Fig. 1). In that group, the BM errors were significantly higher than the other types of error. The CME and normal macula groups had no statistically difference between BM, ILM, and combined errors (Fig. 3).

**Fig. 4:**
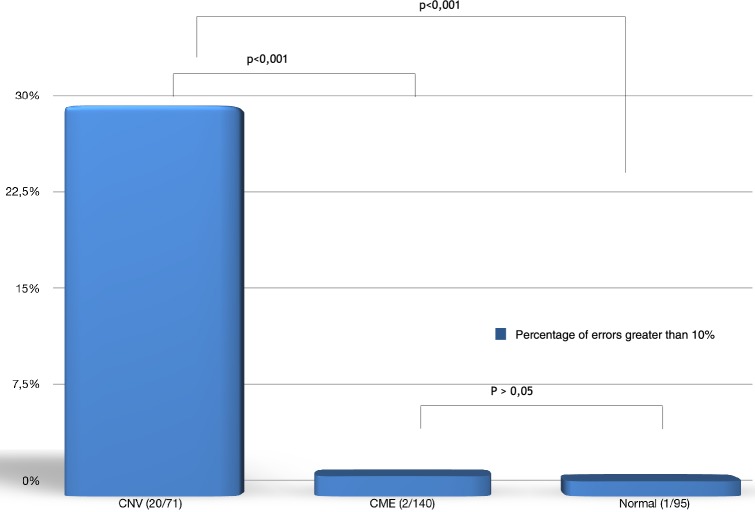
Comparison of the frequency of clinically relevant errors (error greater than 10%) between the automated and manual measurements between the normal macula, the cystoid macular edema (CME), and the choroidal neovascularization (CNV) groups

The frequency of clinically relevant errors (error greater than 10%) between the automated and manual measurements in the central thickness was 1.4% (2 of 140) in the CME group and 28.16% (20 of 71) in the CNV group. Only one error greater than 10% occurred in the normal macula group. The frequency was significantly higher in the CNV group than in the normal macula and CME groups (p < 0.0001). No difference was evident between the normal macula and the CME groups (Fig. 4).

Due to the high prevalence of segmentation error in the CNV group, the QI was checked for correlation with segmentation error in a particular analysis. The average QI in the CNV subgroups, when comparing exams without segmentation error (average QI of 22.66 ± 3.92) and exams with segmentation error (average QI of 21.69 ± 4.7), was similar and had no statistical difference (p = 0.38). In the CNV group, the min QI values in the exams with and without segmentation error were 15.96 ± 4.7 and 18.08 ± 5.68, respectively, with no statistical difference (p = 0.15).

A sub-analysis was performed in the CNV group to correlate the segmentation error location (BM or ILM) with a specific pattern of fluid accumulation. The CNV group was divided in: 1. Absence of fluid, 2. Sub-RPE fluid, 3. Sub-retinal fluid, 4. Intraretinal fluid and 5. Combined fluid, when more than one type of fluid was presente. Using the Fischer Exact Test, no statistically significance (p = 0.999) correlation was found.

## Discussion

The normal human retina is well assessed by OCT scans using a low coherence wave light source and interferometry with a longitudinal resolution of 10 μm [1]. OCT is widely used in clinical trials, but it serves most importantly as a daily tool for clinical decisions in macular diseases. Nevertheless, the assistant ophthalmologist or the ophthalmic technician in charge of the images often misses the manual correction available in the OCT device.

The frequency of segmentation error has been investigated previously in order to predict groups of pathologies associated with higher odds of misalignment of the automated segmentation, which prejudices the interpretation of the OCT scans [6–8]. Our study has demonstrated a difference in central thickness between automatic and manual measurements of 9.80 ± 15.01 μm, which falls within the accuracy expected for the OCT device. The frequency of the segmentation errors was 5.3% (5 of 95) in the normal macula group, which was consistent with the frequencies reported in other studies [9, 10].

Sohn et al. [11] found only one segmentation error in 21 patients with CME subjected to OCT Spectralis analysis, while Lee et al. [12] found only one case in 40 images of 20 patients in a study involving only high quality images. Forte et al. [13] reported 12 cases with segmentation error in 30 patients with diabetic macular edema, including low quality images. Matt et al. found a frequency of moderate and severe mistakes (defined as > 50 μm) of 1.4% in 20 cases with macular edema due to retinal branch venous occlusion [10]. In our study, 140 cases of CME, independent of the cause, were included. Of those, 17.14% presented segmentation errors, and 1.4% were clinically relevant errors (> 10%).

Sadiq et al. [8] found 66% segmentation errors in 29 eyes with wet AMD; more than 50% of these errors were smaller than 10 μm, while 23% of the images had an error over 48 μm. Giani et al. [14], who also used Spectralis OCT, found segmentation errors in 57.6% of 24 eyes with neovascular AMD. Ray et al. [6] evaluated 171 eyes and categorized their fast macular thickness map scan artifacts into six types. Four types (inner and outer retinal misidentification, degraded image artifact, and off-center artifact) were associated with calculation errors. They showed a frequency of artifacts in 43.2% of all their scans. In our study, the frequency of segmentation errors was 66.2% in the CNV group, with 28.16% being higher than 10%. This frequency could influence the interpretation of a therapeutic effect and decision making in clinical and research practice [3–5].

The differences between CME and CNV segmentation error frequencies may reflect a greater distortion of the outer retina and retinal pigment epithelium anatomy in eyes with CNV, as already noted by other authors [7, 9, 15]. Besides the importance of those errors in the follow up of patients, the technology of OCT angiography brings a new dimension to the importance of segmentation errors. The analysis of our findings revealed that more than 50% of the segmentation errors involved the ILM in the CME group, while almost 100% of the errors involved the BM in the CNV group. In the era of the OCT angiography, this could represent the nondetection of retina neovascularization in cases of diabetic retinopathy with retinal edema and nondetection of CNV in cases of RPE distortion. In fact, Lauermann et al. found that segmentation errors affected the analysis of OCT angiography in neovascular AMD in 90% of the cases [16].

Image quality surprisingly did not correlate with segmentation problems. In this study, we did not exclude images with a low QI. Even in the CNV group, which demonstrated a high frequency of segmentation error, a sub-analysis performed by comparing the QI of images with segmentation error (18.08 ± 5.68) versus images without segmentation error (15.96 ± 4.7) revealed no statistical difference (p = 0.15). This finding confirmed that a low quality of the image is not strictly connected to higher rates of segmentation errors. Additionally, the type of fluid accumulation in the CNV group did not correlate with the location of segmentation error, therefore suggesting that the outer retinal distortion caused by the CNV plays a major role in delineation issues.

The goal of this study was to evaluate the impact of segmentation errors on the interpretation of daily practice ophthalmologic exams. We adopted the same criteria that previous studies used to analyze therapeutic efficacy [3, 4], which approaches our findings to the impact of segmentation error in research and real life. Also, we could gather a larger sample size in comparison with other similar publications [7–12, 14]. The presence of the CNV is the major variable that can cause clinically relevant segmentation error (> 10%), thus we strongly recommend that CNV images should be reviewed, especially in clinical trials and when the macular thickness is an important outcome.

Some important limitations of the present study are its retrospective nature and transverse design, as well as the inability to evaluate the impact of segmentation errors over the follow up period, especially in cases that showed higher differences between the automated and manual segmentation. In addition, all exams were performed using the same equipment, and the same results may not be obtained with other devices with different automated segmentation qualities.

## Conclusion

The automated segmentation errors can potentially influence clinical decisions based on macular thickness measurements in eyes with CNV. Conversely, segmentation errors involving CME had rarely (1.4%) enough magnitude to cause misinterpretation of the exam. Therefore, when considering the normal macula, the segmentation errors are likely irrelevant.

## Data Availability

The datasets used and/or analyzed during the current study are available from the corresponding author on reasonable request.

## Abbreviations

AMD: age related macular degeneration
BM: Bruch’s membrane
CNV: choroidal neovascularization
CME: macular cystoid edema
ILM: internal limiting membrane
OCT: optical coherence tomography
QI: quality index
RPE: retinal pigment epithelium

## References

[1] Hee MR, Izatt JA, Swanson EA (1995). Optical coherence tomography of the human retina. Arch Ophthalmol.

[2] Martin DF, Maguire MG, Ying GS, Grunwald JE, Fine SL, Jaffe GJ, CATT Research Group (2011). Ranibizumab and bevacizumab for neovascular age-related macular degeneration. N Engl J Med..

[3] Elman MJ, Aiello LP, Beck RW, Bressler NM, Bressler SB, Edwards AR, Ferris FL, Friedman SM, Glassman AR, Miller KM, Scott IU, Stockdale CR, Sun JK, Diabetic Retinopathy Clinical Research Network (2010). Randomized trial evaluating ranibizumab plus prompt or deferred laser or triamcinolone plus prompt laser for diabetic macular edema. Ophthalmology..

[4] Wells JA, Glassman AR, Ayala AR, Jampol LM, Aiello LP, Antoszyk AN, Arnold-Bush B, Baker CW, Bressler NM, Browning DJ, Elman MJ, Ferris FL, Friedman SM, Melia M, Pieramici DJ, Sun JK, Beck RW, Diabetic Retinopathy Clinical Research Network (2015). Aflibercept, bevacizumab, or ranibizumab for diabetic macular edema. N Engl J Med..

[5] Raman R, Bhende M (2015). Diabetic macular edema. Sci J Med Vis Res Foun.

[6] Ray R, Stinnett SS, Jaffe GJ (2005). Evaluation of image artifact produced by optical coherence tomography of retinal pathology. Am J Ophthalmol.

[7] Song Y, Lee BR, Shin YW, Lee YJ (2012). Overcoming segmentation errors in measurements of macular thickness made by spectral-domain optical coherence tomography. Retina.

[8] Sadiq MA, Rashid A, Channa R, Hatef E, Do DV, Nguyen QD, Sepah YJ (2013). Reliability and reproducibility of spectral and time domain optical coherence tomography images before and after correction for patients with age-related macular degeneration. F1000 Res.

[9] Domalpally A, Danis RP, Zhang B (2009). Quality issues in interpretation of optical coherence tomograms in macular diseases. Retina..

[10] Seibold LK, Kahook MY (2012). The effect of software upgrade on optical coherence tomography measurement of the retinal nerve fiber layer thickness. Middle East Afr J Ophthalmol.

[11] Lee JY, Chiu SJ, Srinivasan PP, Izatt JA, Toth CA, Farsiu S, Jaffe GJ (2013). Fully automatic software for retinal thickness in eyes with diabetic macular edema from images acquired by cirrus and spectralis systems. Invest Ophthalmol Vis Sci..

[12] Forte R, Cennamo GL, Finelli ML, de Crecchio G (2009). Comparison of time domain Stratus OCT and spectral domain SLO/OCT for assessment of macular thickness and volume. Eye..

[13] Matt G, Sacu S, Buehl W, Ahlers C, Dunavoelgyi R, Pruente C, Schmidt-Erfurth U (2011). Comparison of retinal thickness values and segmentation performance of different OCT devices in acute branch retinal vein occlusion. Eye..

[14] Giani A, Cigada M, Esmaili DD, Salvetti P, Luccarelli S, Marziani E, Luiselli C, Sabella P, Cereda M, Eandi C, Staurenghi G (2010). Artifacts in automatic retinal segmentation using different optical coherence tomography instruments. Retina..

[15] Sohn EH, Chen JJ, Lee K, Niemeijer M, Sonka M, Abràmoff MD (2013). Reproducibility of diabetic macular edema estimates from SD-OCT is affected by the choice of image analysis algorithm. Invest Ophthalmol Vis Sci..

[16] Lauermann JL, Woetzel AK, Treder M, Alnawaiseh M, Clemens CR, Eter N, Alten F (2018). Prevalences of segmentation errors and motion artifacts in OCT-angiography differ among retinal diseases. Graefes Arch Clin Exp Ophthalmol.

